# ‘Potentially inappropriate or specifically appropriate?’ Qualitative evaluation of general practitioners views on prescribing, polypharmacy and potentially inappropriate prescribing in older people

**DOI:** 10.1186/s12875-016-0507-y

**Published:** 2016-08-11

**Authors:** Barbara Clyne, Janine A. Cooper, Carmel M. Hughes, Tom Fahey, Susan M. Smith, on behalf of the OPTI-SCRIPT study team

**Affiliations:** 1 HRB Centre for Primary Care Research, Department of General Practice, Royal College of Surgeons in Ireland (RCSI), 123 St. Stephens Green, Dublin 2, Republic of Ireland; 2 School of Pharmacy, Queen’s University Belfast (QUB), 97 Lisburn Road, Belfast, BT9 7BL Northern Ireland

**Keywords:** Potentially inappropriate prescribing (PIP), Older patients, Primary care, Qualitative analysis, Polypharmacy

## Abstract

**Background:**

Potentially inappropriate prescribing (PIP) is common in older people in primary care, as evidenced by a significant body of quantitative research. However, relatively few qualitative studies have investigated the phenomenon of PIP and its underlying processes from the perspective of general practitioners (GPs). The aim of this paper is to explore qualitatively, GP perspectives regarding prescribing and PIP in older primary care patients.

**Method:**

Semi-structured qualitative interviews were conducted with GPs participating in a randomised controlled trial (RCT) of an intervention to decrease PIP in older patients (≥70 years) in Ireland. Interviews were conducted with GP participants (both intervention and control) from the OPTI-SCRIPT cluster RCT as part of the trial process evaluation between January and July 2013. Interviews were conducted by one interviewer and audio recorded. Interviews were transcribed verbatim and a thematic analysis was conducted.

**Results:**

Seventeen semi-structured interviews were conducted (13 male; 4 female). Three main, inter-related themes emerged (complex prescribing environment, paternalistic doctor-patient relationship, and relevance of PIP concept). Patient complexity (e.g. polypharmacy, multimorbidity), as well as prescriber complexity (e.g. multiple prescribers, poor communication, restricted autonomy) were all identified as factors contributing to a complex prescribing environment where PIP could occur, as was a paternalistic-doctor patient relationship. The concept of PIP was perceived to be of variable usefulness to GPs and the criteria to measure it may be at odds with the complex processes of prescribing for this patient population.

**Conclusions:**

Several inter-related factors contributing to the occurrence of PIP were identified, some of which may be amenable to intervention. Improvement strategies focused on improved management of polypharmacy and multimorbidity, and communication across primary and secondary care could result in substantial improvements in PIP.

**Trial registration:**

Current controlled trials ISRCTN41694007

## Background

People over the age of 65 years have a higher prevalence of multimorbidity (commonly defined as the presence of two or more chronic medical conditions), requiring multiple medications (polypharmacy) to manage symptoms and prevent future events.. These combined health care needs, coupled with age-related changes in pharmacokinetics and pharmacodynamics make prescribing in this population a complex task [1–3]. Consequently, evidence indicates that prescribing in older people is often suboptimal, with an increased risk for adverse outcomes including drug-drug interactions, adverse drug reactions and potentially inappropriate prescribing [4, 5]. Potentially inappropriate prescribing (PIP) comprises a number of suboptimal prescribing practices, including inappropriate dose or duration of medication, drug–drug interactions, drug–disease interactions, and use of medications that have a significant risk of an adverse drug event (ADE) [6].

In recent years, PIP in older patients has become an important public health concern, and a significant body of research has emerged on the topic. A recent systematic review identified 36 published tools to assess PIP in older people, the majority of which were explicit (i.e. lists of drugs to avoid) [7]. There is little overlap between the tools in terms of which medications are considered inappropriate but commonalities include the use of long-acting benzodiazepines, the use of tricyclic antidepressants (TCAs), and Non-Steroidal Anti-Inflammatory Drugs (NSAIDs) in combination with other medications (e.g. warfarin) [8]. Quantitative studies have estimated the prevalence of PIP in primary care between 20 - 50 % in older patients, depending on which of these tools has been applied [9–11]. Commonly reported consequences of PIP include increased morbidity, ADEs, hospitalisations, and lower health related quality of life [4, 12, 13]. The most important risk factor for having PIP is polypharmacy [14]. Polypharmacy may describe prescribing of many drugs (appropriately) or too many drugs (inappropriately) [15]. A limited number of randomised controlled trials (RCTs) in primary care have found pharmacist interventions, computerised decision support systems (CDSSs) and multifaceted interventions (involving more than one intervention component) to be somewhat effective in reducing PIP [16].

Relatively few qualitative investigations into the phenomenon of PIP and the processes underlying it have been published to date. A recent meta -synthesis identified seven papers exploring PIP in older people qualitatively, highlighting that some doctors have self-perceived restrictions with regard to prescribing appropriately because of a combination of factors such as patient expectation, tension between experience and guidelines and prescriber fear [17]. The majority of the included studies in this meta -synthesis [17] focused on the experience of prescribing based on single medications (e.g. benzodiazepines) rather than polypharmacy or PIP and only three included perspectives of general practitioners (GPs), highlighting a dearth of information in this area. While it is crucial to determine the prevalence of PIP, the consequences of PIP, and how best to reduce it, it is also essential to explore the perspectives of clinicians in relation to PIP to better inform interventional strategies. The aim of this paper is to explore GP perspectives regarding prescribing and PIP in older primary care patients.

## Methods

### Context and study setting

This study is part of a larger research project conducted in Irish primary care: the OPTI-SCRIPT study (OPTImizing PreSCRIbing for Older People in Primary Care, a clusTer randomized controlled trial). The detailed trial methods and results have been published elsewhere [18–20]. Briefly, this was a cluster RCT involving 21 GP practices and 196 patients which found that a multifaceted intervention was effective in reducing PIP in the intervention group. The intervention comprised: academic detailing with a pharmacist on conducting GP-led medicines review with participating patients; medicines reviews supported by web-based pharmaceutical treatment algorithms for GPs providing evidence-based alternative treatments for specific PIP medicines identified per patient by the research pharmacist (see Appendix for full list [19]); and tailored patient information leaflets. A mixed methods process evaluation was conducted as part of the trial. The process evaluation explored the implementation of the intervention, the experiences of those participating in the RCT, and lessons for future implementation [21]. The qualitative component of this evaluation provided an opportunity to explore aspects of PIP and prescription medication use qualitatively with GPs using a thematic analysis.

### Study population

All interview participants were recruited from the OPTI-SCRIPT study between January and July 2013. The lead study GP in all 21 participating practices, in both the intervention and control arms, was asked to participate in a semi-structured interview, 17 of whom were available for interview (two were on leave and two were unable to schedule an interview within the study time frame).

### Data collection

Data were collected from one to one semi-structured interviews. Semi-structured interviews were the preferred method of data collection as it is a flexible approach, allowing interviewers to alter the sequence of questions or the way in which they are phrased in response to the participants [22]. All interviews were conducted either in person (in a setting of the participants preference) or via telephone. Telephone interviewing is generally used where time or costs are issues, and there is evidence that there is little difference in the quality of the data obtained this way [23, 24]. The interview topic guide covered aspects of the trial evaluation, as well as prescribing related issues. In particular, the following broad sections were pertinent to this current study: reflections on prescribing for older patients in primary care; GPs’ attitudes to the concept of PIP and its perceived relevance in primary care, as presented in Table 1. All interviews were audio recorded (on loud speaker for telephone interviews) and were conducted by one interviewer (BC), a health services researcher with previous experience of conducting qualitative interviews and focus groups.

**Table 1:** Broad prescribing related interview questions

Broad questions	
Could you tell me a little about your experience of prescribing for your older patients *Prompts:* multimorbidity; polypharmacy; patient preference/demands Are you familiar with the concept of PIP or the criteria used to measure it, aside from this study? Could you tell me a little about your perspective on PIP / What’s your view on PIP in primary care? In your opinion, how is PIP important, relevant to practice? What is your opinion of the terminology used, PIP?	

### Data analysis

All interviews were audiotaped and transcribed verbatim by one interviewer (BC). Because the intended analyses focused only on the content of what was said, the interviews were transcribed verbatim (including for example errors in pronunciation/grammar, slang, and emotional expressions such as laughter) but finer details such as brief hesitations or changes in intonation were omitted [25]. All participant data were pseudo-anonymised by assignment of a unique study ID. A thematic analysis was conducted following a six-step process [26]. Two researchers (BC, JAC) independently reviewed the transcripts of the individual interviews several times to familiarise themselves with the relevant data. Small sections of data were assigned a code that summarised the content either. Codes with common features were grouped together in emerging themes, before finally being assigned to overarching themes [26]. In the write up, quotations were used as exemplars of key themes. NVivo 10 was used to assist with organizing the data for analysis.

## Results

### Characteristics of participants

A total of 17 semi-structured interviews were conducted. The majority of GPs were male (*n* = 13, 76 %) and 70 % (*n* = 12) of interviews were conducted in person (Table 2). Compared to a national sample of GPs, a greater proportion of study practices were in urban areas and there were less single-handed practices (Table 2). Three inter-related themes emerged, namely a complex prescribing environment, paternalistic doctor-patient relationship, and relevance of PIP as a concept, and are presented below.

**Table 2:** Characteristics of the interview participants compared to a national sample

Characteristic	OPTI-SCRIPT study N (%)	National estimates^a^ %
Number interviewed	17	476
Male (%)	13 (76.4)	69.0
Urban practice location	12 (70.6)	43.0
Single handed practices	3 (17.6)	35.0
>10 years in practice	14 (82.3)	No estimate
Interview method:		
In person (%)	12 (70.5)	N/A
Telephone (%)	5 (29.5)	
Average interview length (minutes)	14.5 (range 8.56 - 26.31)	N/A

*N/A* Not applicable

^a^ Based on a nationally representative sample of GPs from 2005 [52].

### Complex prescribing environment

Complexity was a major theme encompassing patient, prescriber, and environment factors.

GPs felt that polypharmacy, multimorbidity, and patient heterogeneity, all contributed to complexity at the patient level. Potential side-effects and drug interactions, and perceived poor patient medication adherence, further compounded these difficulties from the GP perspective:

*“Well polypharmacy is the main issue. It’s a huge fear of drug interactions and side effects you know, it’s always a challenge isn’t it? You end up adding more and more tablets to the list and do you take time to review and stop the other ones and all of that?” (GP3)*

In tandem with this, treating complex patients resulted in complexity at the prescriber level, particularly via fragmentation of care and the involvement of multiple prescribers. Multimorbidity resulted in the involvement of multiple prescribers and specialists in patient care, each adding to or amending a patient’s medication list:

*“Yes the eh, constant evolution of a person’s prescription, the lack of stability, there must be a formula somewhere, that would be a nice formula to write, that eh, you know, the complexity of managing your patient’s repeat prescription increases exponentially in relation to the number of specialists they are seeing or something like that.”* (GP13)

Multiple prescriber involvement appeared to impinge on GP prescribing autonomy. GPs reported feeling constrained as they were required to issue repeat prescriptions for patients on the General Medical Services Scheme (GMS) see Table 3, without necessarily having initiated the prescription:

**Table 3:** Key features of the Irish primary health care system

• Mixed public and private funding • Mixed eligibility based on means-testing of income: ○ General Medical Scheme (GMS) card holders (approx. 40 % of population) entitled to free medical visits but pay a small levy for dispensed medications ■ All those aged ≥70 years are automatically eligible GMS since August 2015 ○ Doctor visit card (DVC) holders are entitled to free GP visits but pay for prescriptions in full ○ Private patients pay in full for GP visits and dispensed medications • GPs must transcribe hospital prescriptions to GMS scripts for patients to receive prescription medication for free (subject to prescribing levy) • GPs are self-employed and are contracted by the State to provide services to certain populations (e.g. GMS card holders) • No national register of GPs but it is estimated that there are roughly 2,500 • Routine auditing of prescribing activity does not take place as it does in countries such as the UK, where most prescribers are supported by pharmaceutical advisors

*“Well it limits you eh, very much, the one that stands out there was a lady with [chronic condition], so all of her medication, apart from current illness, was in the hand of the consultant. And of course I had to provide the script through her medical card system, otherwise I probably wouldn’t have seen her because it wasn’t really my prescribing.”* (GP9)

Multiple prescribers and multimorbidity further restricted GP prescribing autonomy in terms of the choice of medications to prescribe when older patients presented to primary care with acute conditions, with GPs feeling constrained in terms of their prescribing options:

*“…the more complex their prescription, the harder it is for me to do my job, almost as if, the more specialist clinics that people are going to, the tighter the straitjacket I’m on. These elderly people who have a lot of symptomatic illnesses as well, you know, attend me, and I have less and less options. If all I had to do was just care about the person’s headache, or their back pain, or their pneumonia, I could just do what I would normally do. But because they are seeing 3 different specialists and because they are on 3 different suites of medicines, you know, I have to cognisant of… You know I’m not complaining about that, it’s just a fact that it does get difficult. You’ve less and less wiggle room you know.”* (GP13)

Patient and prescriber complexity contributed to a prescribing environment which was characterised as complex and isolating:

*“The complexity of prescribing for the elderly is a lonely game.”* (GP5)

Within this environment, one of the biggest contributing factors to the occurrence of PIP and general prescribing errors was poor communication. Communication between primary and secondary care was identified as problematic in both directions. However, for the study participants, the most salient issue was that changes made in hospital/outpatient settings were often not communicated in a timely manner to inform decision making:

*“The real difficulty that we have, ok, is that we are basically transcribing hospital prescriptions in a lot of cases onto GMS [General Medical Services Scheme] scripts, ok, and a lot of the time when we get the prescriptions, it’s from an outpatient clinic. It might be in good cases 2 weeks later, in other cases, 5 or 6 months later when we get a letter of explanation for why the changes were made, ok, so do we ignore the prescription until we get an explanation for it?”* (GP21)

### Paternalistic doctor-patient relationship

Within a paternalistic doctor-patient relationship, the doctor is viewed as the authority figure, acting in the best interest of patient, while the patient cooperates [27]. In general, GPs in this study characterised themselves in terms of this type of model. Some GPs clearly adopted an authoritarian/parental approach to encounters with older patients:

*“I mean they will take my lead on this, of course. I mean if this is what I tell them, em, they will say ‘Yes doctor, of course’.” (GP9)*

*“Generally the older patients do as I asked them to do, and what I mean by compliance is that they mix up their tablets, but luckily, our local pharmacy now vacuum pack them for them…I mean I know, it sounds like baby stuff but to be honest with you, we have actually gone back to as if you were telling children when they have to take everything, and it works really well.” (GP23)*

However, it is unclear if this paternalistic model was employed by choice, or if GPs felt compelled to take responsibility where patients adopted a more passive approach to their medication management:

*“I mean it certainly amazes me how blindly they will take increases in their medications, it’s incredible you know, eh and you know, ‘Oh I’m going to increase your blood pressure tablet by one, I’m going to add another anti-cholesterol tablet, O you’re a little bit sleepless we’ll add another…’, you know, they could walk out of here with three new tablets on their eh, script, they just accept it.”* (GP2)

This passive approach may be a consequence of the complex medication regimen many patients had, without fully understanding which medications they were taking and why:

*“…sometimes, people are on tablets and they don’t know why and the reason that they are on them has long since passed”* (GP22)

GPs also frequently reported that poor communication was problematic in this patient group, requiring GPs to take extra measures to get accurate information on patient adherence:

*“Because sometimes, if a tablet is upsetting them, some of them* [patients] *can be embarrassed to tell you, and they just don’t take them, and they end up with a stock pile, so I ask them to bring everything in.”* (GP23)

### Perceived value of PIP concept

Most GPs accepted that inappropriate prescribing was occurring in primary care in these complex patients:

*“I’ve no doubt that there is, that we do prescribe inappropriately. Or that patients are inappropriately kept on medications for long periods when, in fact, that medication may have only been prescribed, or intended for a shorter term, a shorter duration.”* (GP26)

Many GPs were not, however, aware of PIP as a concept and the published criteria such as the Beers criteria and STOPP (Screening Tool of Older Person’s Prescriptions) criteria were not often utilised in routine practice. While the GPs interviewed were receptive to receiving advice on prescribing and were keen to improve where possible, a number were sceptical of the utility of such criteria when it came to treating such a heterogeneous population:

*“I think in the age group we are prescribing for, my X plus patients who are over 70, are all individuals. They are individuals themselves, but in fact, they are all on different cohorts of medications. So I don’t think you can be, when you look at best practice in prescribing one agent, you really have to say best practice in prescribing one agent with maybe, anti-coagulants and maybe, anti-hypertensives, and maybe, eh, multiple inhalers, and analgesics at the same time.”* (GP5)

Furthermore, a tension existed between specific criteria, and the risk-benefit of altering medications in the context of individuals. Where the perceived risk of continuing a PIP was seen as lower than the potential complications from altering a medication, GPs were reluctant to make changes:

*“Sometimes, however, for example, in relation to benzodiazepine, em, you know, somebody might be on benzodiazepines and has been for 40 years, which one of the patients actually was, I don’t think it’s appropriate to stop that. If they’re stable and they can get on with their lives then I think it would cause more hassle for them and so on and so forth, so, what might be generally inappropriate might be specifically appropriate.”* (GP1)

The term ‘potentially inappropriate prescribing’ evoked mixed reactions in the GPs, with six of them reporting that they found the term particularly negative, value-laden and accusatory and did not incorporate the difficulties of prescribing for older patients faced by the GPs, as outlined above:

*“Inappropriate connotes carelessness, and I think very few of us are careless…”* (GP5)

The remaining GP interviewees did not express any negative feelings towards the terminology or find it to be problematic:

*“Potentially inappropriate prescribing, I think that’s quite good, actually, because it’s, it’s potentially. It’s not giving you an absolute.”* (GP22)

## Discussions

### Summary

This study highlights some GPs’ perspectives on PIP and polypharmacy in older patients. Three inter-related themes emerged, namely a complex prescribing environment, paternalistic doctor-patient relationships, and a limited relevance of the PIP concept for GPs. GPs highlighted that prescriber (e.g. multiple prescribers) and patient (e.g. multimorbidity) factors, and characteristics of a paternalistic doctor-patient relationship, contributed to a complex environment in which potentially inappropriate prescribing could occur. However, the utility of the PIP concept, and the criteria to measure it, was questioned by GPs as it often failed to take this complexity into account.

### Comparison with existing literature

Consistent with previous qualitative explorations, this study has highlighted that multiple, inter-related factors influence PIP [17, 28]. Polypharmacy, multimorbidity, and fragmentation of care have all been identified as contributing to a complex prescribing environment [17, 28, 29]. These factors are common risk factors for PIP, some of which may be amenable to intervention. At the patient level, polypharmacy is one of the most consistently reported predictors of PIP [10, 30, 31]. Appropriate polypharmacy may be improved through a number of interventional strategies such as the involvement of pharmacists in pharmaceutical care and strategies focusing on deprescribing [32–34]. However, the evidence base supporting the mechanisms of how to deprescribe is limited [35]. Improving the management of patients with multimorbidity through multifaceted interventions targeted at risk factors or specific functional difficulties may potentially improve prescribing, however, the evidence to date is limited [36]. At the prescriber level, the number of prescribers involved in patient care has been identified as an important predictor of PIP [37, 38]. Our findings suggest that fragmentation of care between multiple prescribers results in poor communication of up-to-date patient medication information - a finding which resonates with previous qualitative investigations on treating patients with multimorbidity [29, 39]. Improving communication systems throughout a healthcare system is likely to be a difficult task as it represents a larger system/policy level change. Improving medicines reconciliation represents one feasible mechanism to decreasing medication errors at transitions of care [40].

Over the last century, the conceptualisation of the doctor-patient relationship has altered, moving towards models of shared decision making [27, 41, 42]. Consistent with previous qualitative findings in a hospital population [43], our study provides evidence for the continued existence of a paternalistic doctor-patient relationship in older patient groups. However, GPs may view this relationship structure as necessary due to patient confusion with their complex medication regimens, a passive approach to medication management, and/or attempts to preserve their prescribing autonomy [44]. Promoting involvement of older patients in prescribing decisions, or shared decision-making, may impact on PIP, as exemplified by the recent findings of the EMPOWER trial [45]. This study found that a patient educational tool aimed at engaging older adults in discussing the harms of benzodiazepine use with their physician and/or pharmacist, resulted in significant reductions in benzodiazepine use [45]. However, shared decision-making in this group is likely to be complicated by polypharmacy, the involvement of multiple prescribers, and patient knowledge of medication which is often poor in this group [46, 47]. GPs reported feeling restricted in terms of prescribing autonomy with the choice of treatment often limited by prescriptions from specialist clinics. In circumstances like this, shared decision-making regarding prescription medication use is likely to be difficult and restricted in scope.

Consistent with previous qualitative findings [28, 48], GPs in this study reported a limited knowledge of PIP and published PIP criteria. GPs accepted that PIP may be occurring, however, there was disagreement between these participants as to how useful it was to refer to it as such. A number of GPs found the terminology adversarial and critical. As well as questioning the terminology, a number of GPs were critical of PIP criteria, highlighting that it failed to adequately take into consideration the complexity of an older heterogeneous patient profile, a common criticism of such criteria. Consequently, the criteria are often perceived to be limited in terms of clinical utility and relevance in primary care [49, 50]. In complex patients perceived as stable, GPs have been found to prefer to ‘maintain the status quo’, sometimes referred to as clinical inertia [28, 51]. Our result confirmed this, highlighting that particularly in the cases of medications such as benzodiazepines, where the risk of stopping the medication is perceived as worse than continuing it, GPs did not find PIP criteria useful. To be effective, such tools need be applicable in routine clinical practice, not only in a research environment. However, as our study indicates, this gap may not have yet been successfully bridged in primary care.

### Strengths and limitations

This study is an important contribution to the limited qualitative data in this area. The qualitative methodology used allowed an exploration of the complexity of PIP in primary care not accessible in the numerous quantitative, prevalence studies in the current literature. However, the present study has a number of limitations. These interviews were conducted as part of the OPTI-SCRIPT trial process evaluation, which limits the findings in two main ways. Firstly, the process evaluation was designed to explore elements of intervention implementation and study participation. Therefore, the topic guide was not designed to explore the issues outlined in this paper solely, and interviews were shorter on average (14 minutes) than may be expected to explore prescribing as a topic. While the themes presented here emerged strongly from the data, arguably, a more detailed interview on prescribing and prescription medication use alone may have produced a more nuanced account. Secondly, the sample was limited to only those GPs who participated in the OPTI-SCRIPT trial. While this resulted in a good response rate, the participants may not be representative of GPs nationally. Study participants were predominantly male, and urban based and as volunteers to this study, may be more informed about PIP than the general population. However, when compared to a national sample of GPs, the participants were broadly similar. Overall, d our results are consistent with the limited number of qualitative studies in this area, increasing confidence in the findings.

## Conclusions

PIP is common in older populations in primary care. This study identified several inter-related factors that contribute to the occurrence of PIP including a complex prescribing environment, paternalistic doctor-patient relationships and limited relevance of the PIP concept for GPs. Several of these have been highlighted as amenable to intervention, including improved management of polypharmacy and multimorbidity and decreasing PIP. However, there is a need for stronger evidence of the effectiveness of such interventions in resulting in clinically significant patient improvements in patient primary care [16, 34, 36]. The concept of PIP is perceived to be of limited usefulness to GPs and the criteria to measure it may be at odds with the complex processes of prescribing for this patient population. Future strategies for improving PIP need to be cognisant of such barriers.

## Abbreviations

CME, continuing medical education; GMS, General Medical Services; HRB, Health Research Board; OPTI-SCRIPT, OPTImizing PreSCRIbing for Older People in Primary Care, a clusTer randomised controlled trial; PILs, patient information leaflets; PIP, potentially inappropriate prescribing; PPI, proton pump inhibitor; RCT, randomised controlled trial

## Appendix

**Table 4:** Selected Prescribing Criteria/Prescribing Indicator [16]

Criteria	Concern	Estimated prevalence in Ireland^a^
PPI for peptic ulcer disease at full therapeutic dosage for > 8 weeks	Earlier discontinuation or dose reduction for maintenance/ prophylactic treatment of peptic ulcer disease, oesophagitis or GORD indicated	4.1- 16.7 %
NSAID (>3 months) for relief of mild joint pain in osteoarthritis	Simple analgesics preferable and usually as effective for pain relief	1.1 - 8.8 %
Long-term (i.e. >1 month), long-acting benzodiazepines e.g. chlordiazepoxide, flurazepam, nitrazepam, chlorazepate and benzodiazepines with long-acting metabolites e.g. diazepam	Risk of prolonged sedation, confusion, impaired balance, falls	3.0-9.1 %
Any regular duplicate drug class prescription e.g. 2 concurrent opiates, NSAIDs, SSRIs, loop diuretics, ACE inhibitors. Excludes duplicate prescribing of drugs that may be required on a PRN basis e.g. Inhaled beta 2 agonists (long and short acting) for asthma or COPD, and opiates for management of breakthrough pain	Optimisation of monotherapy within a single drug class should be observed prior to considering a new class of drug	2.2 – 6.0 %
TCAs with an opiate or calcium channel blocker	Risk of severe constipation	0.4-2.0 %
Aspirin at dose >150 mg/day	Increased bleeding risk, no evidence for increased efficacy	0.1-1.%
Theophylline as monotherapy for COPD/Asthma	Risk of adverse effects due to narrow therapeutic index	0.6-1.2 %
Use of aspirin and warfarin in combination without histamine H_2_ receptor antagonist or PPI	high risk of GI bleeding	0.3-1.1 %
Doses of short-acting benzodiazepines, doses greater than: lorazepam 3 mg; oxazepam 60 mg; alprazolam 2 mg; temazepam 15 mg; and triazolam 0.25 mg	Total daily doses should rarely exceed the suggested maximums	1.0-1.5 %
Prolonged use (>1 week) of first generation antihistamines i.e. diphenydramine, chlorpheniramine, cyclizine, promethazine	Risk of sedation and anticholinergic side effects	<1.0 %
Warfarin and NSAID together	Risk of GI bleeding	0.7-1.7 %
Calcium channel blockers with chronic constipation	May exacerbate constipation	<1.0 %
NSAID with history of peptic ulcer disease or GI bleeding, unless with concurrent histamine H_2_ receptor antagonist, PPI or misoprostol	Risk of peptic ulcer relapse	<1.0 %
Bladder antimuscarinic drugs with dementia	Risk of increased confusion, agitation	<1.0 %
TCAs with constipation	May worsen constipation	<1.0 %
Digoxin at a long-term dose > 125 μg/day (with impaired renal function)	Increased risk of toxicity	<1.0 % <1.0 %
Thiazide diuretic with a history of gout	May exacerbate gout	<1.0 %
Glibenclamide (with type 2 diabetes mellitus)	Risk of prolonged hypoglycaemia	<1.0 %
Aspirin with a past history of peptic ulcer disease without histamine H2 receptor antagonist or PPI	Risk of bleeding	<1.0 %
Prochlorperazine or metoclopramide with Parkinsonism	Risk of exacerbating Parkinsonism	<1.0 %
TCAs with dementia	Risk of worsening cognitive impairment	<1.0 %
TCAs with glaucoma	Likely to exacerbate glaucoma	<1.0 %
TCAs with cardiac conductive abnormalities	Pro-arrhythmic effects	<1.0 %
Long-term corticosteroids (>3 months) as monotherapy for rheumatoid arthritis or osteoarthritis	Risk of major systemic corticosteroid side-effects	<1.0 %
Bladder antimuscarinic drugs with chronic prostatism	Risk of urinary retention	<1.0 %
NSAID with heart failure	Risk of exacerbation of heart failure	<1.0 %
TCAs with prostatism or prior history of urinary retention	Risk of urinary retention	<1.0 %
Systemic corticosteroids instead of inhaled corticosteroids for maintenance therapy in COPD/Asthma	Unnecessary exposure to long-term side-effects systemic steroids	<1.0 %
Bladder antimuscarinic drugs with chronic glaucoma	Risk of acute exacerbation of glaucoma	<0.01 %
NSAID with SSRI	Increased risk of GI bleed	N/A
Bladder antimuscarinic drugs with chronic constipation	Risk of exacerbation of constipation	N/A
Prednisolone (or equivalent) > 3 months or longer without bisphosphonate	Increased risk of fracture	N/A
NSAID with ACE-inhibitor	Risk of kidney failure, particularly if presence of general arteriosclerosis, dehydration or concurrent use of diuretics	N/A
NSAID with diuretic	May reduce the effect of diuretics and worsen existing heart failure	N/A

Abbreviations – *ACEI* angiotensin-converting-enzyme inhibitor, *COPD* chronic obstructive pulmonary disease, *GI* gastro-intestinal, *NA* not available, *GORD* gastro-oesophageal reflux disease, *NSAID* Nonsteroidal anti-inflammatory drug, *PPI* Proton Pump Inhibitor, *PRN* Pro re nata, as needed, *SSRI* Selective serotonin reuptake inhibitor, *TCA* Tricyclic Anti-depressant

^a^Prevalence – the proportion of the study population with 1 or more potentially inappropriate medications from the literature
